# In Vivo Evaluation of Laser-Textured Air Plasma in Osseointegration of Dental Implants

**DOI:** 10.3390/ma18163810

**Published:** 2025-08-14

**Authors:** Larissa Azeredo da Silva Lessa Nicolau, Suelen Cristina Sartoretto, Pamella Santana Nunes, Ezio Gheno, Jose Mauro Granjeiro, Domenico D’Angelo, Federico Mussano, Monica Diuana Calasans-Maia, Olivio Della Bella, Francesca Motta, Rafael Seabra Louro

**Affiliations:** 1 Post-Graduation Program in Dentistry, Fluminense Federal University, Niteroi 24020-140, Brazil; larissalessanicolau@gmail.com (L.A.d.S.L.N.); pamellasn@id.uff.br (P.S.N.); 2 Clinical Research Laboratory, Dentistry School, Universidade Federal Fluminense, Niteroi 24220-140, Brazil; susartoretto@hotmail.com (S.C.S.); jmgranjeiro@gmail.com (J.M.G.); 3 Oral Surgery Department, Fluminense Federal University, Niteroi 24220-140, Brazil; drrafaelseabra@gmail.com; 4 Department of Surgical Sciences and Integrated Diagnostics, University of Genova, 16132 Genova, Italy; eziogheno@gmail.com; 5 National Institute of Metrology, Quality and Technology (INMETRO), Duque de Caxias 25250-020, Brazil; 6 Plasma Nano-Tech Lab, Environment Park S.p.A., 10144 Turin, Italy; domenico.dangelo@envipark.com; 7 Bone and Dental Bioengineering Laboratory, CIR Dental School, Department of Surgical Sciences, University of Turin, 10126 Turin, Italy; federico.mussano@unito.it; 8 Biomec Srl, 23823 Colico, Italy; odellabella@biomec.net (O.D.B.); fmotta@biomec.net (F.M.)

**Keywords:** osseointegration, dental implants, rabbits, histomorphometric evaluation

## Abstract

The different macro and micro geometries of dental implants are parameters that directly affect osseointegration, making them an important area for research. The objective of this preclinical study was to compare, through histological and histomorphometric analyses, the biological response of two different dental implant surfaces in osseointegration. Surface morphology and chemistry were characterized by SEM/EDX, optical-emission spectroscopy, protein adsorption (BSA), and adipose-derived stem-cell morphology. For the in vivo arm, ten commercially pure titanium implants (n = 5 LS160 + 5 SBAE) were placed bilaterally in the tibiae of five skeletally mature New Zealand rabbits (one implant of each surface per animal). After six weeks, undecalcified sections were prepared and bone-to-implant contact (BIC) and bone-area-fraction occupancy (BAFO) were quantified histomorphometrically. Data normality was confirmed with the Shapiro–Wilk test; paired two-tailed Student’s *t*-tests were applied (α = 0.05). Results: The descriptive histological analysis showed a fraction of pre-existing bone in all experimental groups, which probably ensured primary stability. Adjacent to this area, it was possible to observe peri-implant newformed bone in all tested groups. The results of the histomorphometric analysis of BIC and BAFO were considered normal by the Shapiro–Wilk test (*p* > 0.05); after six weeks of implantation, the BIC values for the LS160 and SBAE groups were 44.13 (15.83–72.43) and 39.24 (10.72–89.21), respectively. The analysis of variance (ANOVA and Tukey’s post-test) showed no statistical differences between the groups tested. Likewise, the bone volume density showed no statistical differences between the groups (ANOVA and Tukey’s post-test) with averages of 41.27 (C.I. 24.00–58.55) and 26.52 (C.I. −17.51–70.54) in the LS160 and SBAE groups, respectively. Although both surfaces showed similar osseointegration after six weeks, the new surface appears to be a promising, eco-friendly alternative to SBAE. Future studies with shorter time points and larger samples are needed to assess early biological responses.

## 1. Introduction

The use of dental implants in a safe, predictable, and efficient manner allows for aesthetic and functional dental rehabilitation [1]. The biocompatibility and superior mechanical properties of titanium stand out, making it the material of choice [2,3], as it permits osseointegration, i.e., the biological process by which a bone forms a direct, structural, and functional connection with the surface of an implant without any intervening soft tissue. Clinically, osseointegration is defined as an asymptomatic process entailing a rigid fixation between alloplastic materials and bone during function, sometimes known as secondary stability [4].

Several factors influence the achievement of osseointegration, including the bone condition, the surgical technique performed, and the composition and features of the implant material along with its functional loading [2,5]. No single factor guarantees dental implant survival, as everyone has unique characteristics regarding the quality of bone tissue, masticatory strength, and distribution [6]. Decades ago, however, the surface properties of the implants, such as roughness, acquired growing attention because rough surfaces outperformed smooth ones (the so-called machined surfaces) [7].

Hence, a flurry of surface treatments increasing surface roughness were introduced, among which the following can be mentioned: acid etching, sandblasting, anodization, titanium plasma spraying, and calcium phosphate coatings [8]. The acid treatment guarantees a smoother surface, while sandblasting provides greater resistance to implant removal [9]. Double acid etching is admitted as having greater osseointegration forces when compared to machined surfaces. However, it is suggested that it reduces fatigue resistance and causes microcracks, damaging the physical properties of titanium. Furthermore, when comparing single and double acid etching, there is no relevant difference in the wetting angle, roughness, or removal torque [10].

A combination of sandblasting and acid etching and double acid etching are both widely adopted clinically, as is the anodization process that allows for the formation of a thicker titanium oxide layer than other subtractive technologies. Overall, dental industries prefer the subtractive surface modification techniques to the additive ones, which may be hindered by delamination and chipping. Under this perspective, in the last few years, laser texturing has acquired interest as a possible alternative to other subtractive treatments, since it does not involve the manipulation of possibly harmful chemical reagents like strong acids, and it allows for strict control of the roughening pattern that may become non-stochastic [11].

Surface topography, quantitatively expressed as roughness measurements, cannot be separated theoretically from other descriptors such as the surface free energy dictating the wettability properties of a given surface. Based on the remarkable in vitro effects of enhancing cell adhesion, several authors have increased surface wettability through physical methods like UV light [12] and cold plasma technology [13]. Sound preclinical evidence was achieved in an animal model for UV functionalization [14]. The biomechanical characteristics and histomorphometric properties demonstrated in preclinical studies clarify the advantage of modifications to the surfaces of dental implants [15]. The literature infers the need for further studies to define the ideal properties for the surfaces of dental implants and for them to be standardized to ensure reliable results and predictability [16]. In vivo studies are recommended to confirm the potential biological benefits of plasma treatment [17]. Likewise, regarding the use of lasers for implant surface modification, it is difficult to obtain a reliable comparison due to the different techniques, configurations, and high variability of laser types, recognizing the standardization of studies [18].

This study, therefore, aimed to compare the biological response elicited by a clinical standard surface (sandblasted and acid etched) with a new surface obtained by the combination of laser texturing and air-based plasma treatment, both in vitro using a mesenchymal stem cell model and in vivo through histomorphometric evaluation, six weeks after implantation in rabbits. The null hypothesis was that there was no difference between the test and the control surfaces in the cell response and osseointegration process.

## 2. Materials and Methods

Two different surface modifications were implemented on planar titanium disks and implants for animal usage: a laser-textured oxygen plasma-treated surface (henceforth named LS160) and a standard sand-blasted acid-etched surface (henceforth called SBAE). LS160 was obtained through laser radiation from a nano or femto fiber source, which ablated the surface and assumed a pre-established texture. Subsequently, the surfaces were treated with an ionizing plasma beam at atmospheric pressure using air as the process gas, so that a titanium oxide layer up to 30 nm thick was formed on the surface. The plasma treatment was performed using an OpenaAir^®^ PFW1004 plasma jet system from Plasmatreat GmbH (Steinhagen, Germany). The generator was the FG5001, with a supply voltage of 100–240 V. The system is equipped with a Plasma Control Unit that allows for high performance with precise regulation of plasma power, ionizing gas flow, and control functions. One hundred percent of the reference voltage was used, resulting in minimal electrical pulsation, ensuring a stable discharge at 21 kHz–16 A. The Plasma Jet system was equipped with the RD1004 rotary nozzle (Plasmatreat GmbH, Steinhagen, Germany) to generate cold plasma. The pressure of the ejected gas was 0.25 mbar. For SBAE, the roughening process was attained through sandblasting with coarse-grained sand (250–500 m in diameter) followed by washing with deionized water in an ultrasonic tank and air drying. Subsequently, the surface was immersed into a solution containing sulfuric acid and hydrogen peroxide (3:1). After thoroughly washing in deionized water, the specimens were dried with hot air and treated superficially with argon plasma.

Commercially pure titanium (grade IV) specimens were machined to obtain 8 mm × 3 mm cylinders (2r × h) and treated with the two surface treatments for the in vitro tests, while a total of ten dental implants were allocated to the animal experimentation: five LS160 (Group 1) and five SBAE (Group 2). All the specimens, both for in vivo and in vitro tests, were sterilized with gamma radiation according to ABNT NBR 15729:2009 [19]. One implant from each experimental group was installed in the tibia region of five rabbits, totaling two implants in each animal, according to the information and guidelines of the implant system manufacturer.

### 2.1. Optical Emission Spectroscopy

Optical Emission Spectroscopy (OES) is a fundamental technique for analyzing the optical emissions of excited species within the plasma. Specifically, during the phase of studying plasma parameters such as power, ionizing gas volume, and gas type, OES is essential for studying plasma kinetics and chemistry, helping in the optimization of plasma parameters for various applications. In this study, to examine the species spectra in the sensitive range from 150 to 1100 nm as the wavelength, an Ocean Optics LIBS2500 Plus spectrometer (Ocean Optics, Inc., Dunedin, FL, USA), a fiber optic cable, and Oceanview software (version 2.0.8) were used. OES was performed before investigating the effects of plasma on the surface of titanium disks. The probe was positioned 10 mm away from the plasma jet nozzle. In this specific case, the intensity of the peaks related to the oxygen species present in both molecular and atomic radical forms was observed.

### 2.2. Scanning Electron Microscopy and EDX Analysis

The microstructure was studied by means of a Scanning Electron Microscope (Phenom XL G2 Desktop SEM, Thermo Fisher Scientific, Waltham, MA, USA). Before examination, the samples were washed in ultrapure water, rinsed thoroughly in a 70% ethanol water solution, cleaned ultra-sonically in absolute ethanol for 20 min, and finally air dried under a chemical hood. EDX analyses were performed on the samples for quantification of the atomic composition (Phenom XL G2 Desktop SEM, Thermo Fisher Scientific, Waltham, MA, USA).

### 2.3. Protein Adsorption

A 5% solution of Bovine Serum Albumin (BSA) in Phosphate-Buffered Saline (PBS) was adsorbed onto the samples by incubation at 37 °C for 20 min. Samples were then washed twice with PBS, and the adsorbed protein was eluted with Tris Triton buffer (10 mM Tris (pH 7.4), 100 mM NaCl, 1 mM EDTA, 1 mM EGTA, 1% Triton X-100, 10% Glycerol, and 0.1% SDS) for 10 min. A Pierce™ BCA Protein Assay Kit (Life Technologies, Carlsbad, CA, USA) was employed to quantify calorimetrically the amount of adsorbed protein according to the manufacturer’s instructions.

### 2.4. Cellular Morphology

To study the cellular morphology of the specimens, adipose stem cells ASC52hTert (ATCC) were maintained in Alpha-MEM (Life Technologies, Milano, Italy) with 10% FBS, 100 U/mL of penicillin, and 100 μg/mL of streptomycin in a humidified atmosphere at 37 °C. Cells were seeded on the samples at a concentration of 7000 cells/sample in a 48-well plate (BD, Franklin Lakes, NJ, USA). After 24 h, the samples were washed in PBS and fixed with 4% paraformaldehyde in PBS for 10 min. After washing with PBS, cells were permeabilized with 0.1% Triton X-100 (Sigma-Aldrich, Milano, Italy) in PBS, washed again in PBS, and incubated for 30′ with Image-iT™ FX Signal Enhancer (Thermo Fisher Scientific Inc., Waltham, MA, USA). Cells were stained with Alexa 488-Phalloidin (Life Technologies, Milano, Italy) to detect the cytoskeleton and mounted in a ProLong™ Gold Antifade Mountant with DAPI (Thermo Fisher Scientific Inc.). Images were acquired with a Nikon Eclipse Ti-E microscope using different objectives: Nikon Plan 4× and 20× (Nikon Instruments, Amsterdam, The Netherlands).

### 2.5. In Vivo Study

The Ethics Committee for Animal Use of the Fluminense Federal University (CEUA-UFF) approved this research under protocol number 5771310123 (attached) and followed the Brazilian Guideline for the Care and Use of Animals for Scientific and Educational Purposes—DBCA and the Guidelines for the Practice of Euthanasia, both from CONCEA, as well as the guidelines of ARRIVE [20] and PREPARE [21] regarding the relevant items.

This research was performed in accordance with the guidelines of the 3Rs Program (Reduction, Refinement, Replacement), which aims to reduce the number of animals during experimentation and minimize pain and discomfort [22].

The sample size was calculated according to a previous study [23] with the online platform Sealed Envelope (https://www.sealedenvelope.com/power/continuous-superiority/, access date: 1 February 2023). In that study, the primary endpoint of BAFO after 28 days was 34.3% (±6.26) for the control group. Considering a significant 40% BAFO effect after 6 weeks, the calculated sample size was five animals per group at a 5% significance level and 90% power (1 beta).

### 2.6. Animal Maintenance

This study used five healthy and skeletally mature male and female New Zealand white rabbits at least four months old and weighing between 3 and 4 kg. The animals were kept at the Animal Experimentation Laboratory located at the Fluminense Federal University (Niteroi, Rio de Janeiro, Brazil).

Before and after the experimental period, the animals were kept in a Rabbitat Rack for Rabbits with five mini-isolators (ALESCO^®^, São Paulo, Brazil) measuring 2210 × 950 × 1795 mm (1 animal/mini-isolator). They were fed a standard diet consisting of pelleted feed (Coelhil^®^, Socil, São Paulo, Brazil) at 150 g/day at regular intervals and portions and water *ad libitum* through a stainless-steel nipple drinker. The water and feed were changed daily to prevent the formation and proliferation of fungi due to prolonged exposure to the food. The room temperature was maintained between 16 and 20 °C, as this is ideal for the animals’ growth, and the photoperiod control of 12 to 12 h of light and dark was established to provide the correct metabolic cycle. The animals were monitored and anesthetized by an experienced veterinarian.

### 2.7. Anesthesia and Surgery Procedures

All procedures that could result in anxiety and/or pain were performed under general anesthesia. The animals were weighed on a precision scale and were not deprived of food or water before the procedure to avoid hypoglycemia.

The drugs used in the pre-anesthetic medication were Ketamine Hydrochloride 10% (20 mg/kg) (Cetamin^®^, Syntec do Brasil Ltd.a., São Paulo, Brazil) and Xylazine Hydrochloride 2% (1 mg/kg) (Xilazin^®^, Syntec do Brasil Ltd.a., São Paulo, Brazil) mixed in the same syringe and administered intramuscularly in the right quadriceps femoris. Then, Tramadol Hydrochloride (4 mg/kg) (União Química^®^, São Paulo, Brazil) was administered intravenously.

**Figure 1:**
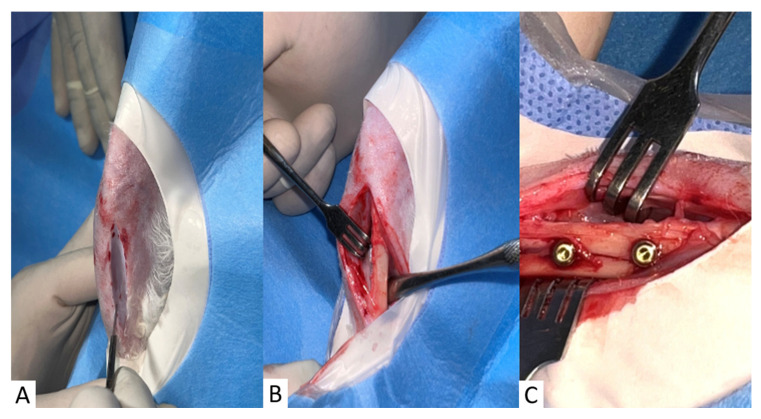
(**A**) Right tibia after degermation, antisepsis, infiltration with local anesthetic, and surgical incision. (**B**) Dissection of soft tissues and exposure of the tibia. (**C**) Dental implants placed.

Two dentists previously trained in rabbit tibial surgery performed all surgical procedures. After anesthetization and monitoring, each animal was positioned in dorsal decubitus, and the tibia region was shaved. Degerming was performed with a 2% chlorhexidine degerming agent (Rioquímica S.A, São Paulo, Brazil), followed by rinsing with a sterile saline solution (Fresenius Kabi Brasil Ltd.a., Ceará, Brazil) and drying with sterile gauze (AMED S/A, Minas Gerais, Brazil). After that, 1.8 mL of 3% prilocaine hydrochloride with felypressin 0.03 IU/mL (DFL^®^, DFL Indústria e Comércio S.A., Rio de Janeiro, Brazil) was administered by infiltration along the tibial incision site. Then, a sterile, fenestrated surgical field was positioned to isolate the surgical region. An incision was made in the skin up to the skeletal plane, allowing surgical access to the tibial region. After incision, undermining, and exposure of the tibia, two perforations measuring 3 mm were made with a sequence of drills from the Oxy Implants kit, recommended by the manufacturer, coupled to a contra-angle (Kavo^®^, Sprimont, Belgium) in a low-speed motor (1200 RPM) (BLM 600 PLUS, VK Driller^®^, São Paulo, Brazil) intermittently, with profuse irrigation of 0.9% sodium chloride solution (Fresenius Kabi Brazil Ltd.a., Ceará, Brazil) to avoid tissue necrosis (Figure 1).

The installation of the implants was randomized so that, in each animal, a different sequence was used from superior (proximal end of the tibia) to inferior (distal end of the tibia). One LS160 implant and one SBAE implant were placed in the same tibia of each animal.

After implantation, the periosteum and skin were sutured using a simple suture on the periosteum and a continuous suture on the skin using Mononylon Ethilon 4.0 thread (ETHICON^®^, Johnson & Johnson do Brasil Indústria e Comércio de Produtos para Saúde Ltd.a, São Paulo, Brazil). The surgical wound was left uncovered. The animals received Meloxicam (0.3 mg/kg) (Eloxicam 0.2%^®^, CHEMITEC Agro-Veterinária, São Paulo, Brazil) in a single dose every 24 h for three days via the intramuscular route.

After the surgical procedure, each animal remained under observation inside the mini-isolators for two hours and, after recovering from anesthesia, received food and water ad libitum. The protocols for anesthesia and surgical procedures were based on methodologies previously established and validated in earlier studies [23,24,25,26,27].

### 2.8. Sample Collection

Six weeks after surgery, the animals were weighed on a precision scale and anesthetized with Ketamine Hydrochloride (2 mg/kg) (Cetamin^®^ Syntec do Brasil Ltd.a., São Paulo, Brazil) and Xylazine Hydrochloride (10 mg/kg) (Xilazin^®^ Syntec do Brasil Ltd.a., São Paulo, Brazil) administered intramuscularly, and then an overdose of Isoflurane (Isoforine^®^ Cristália, São Paulo, Brazil) above 10%, leading to death. A new incision was made at the first surgery site to collect the specimens and access the three implantation sites using the same instruments described. Using piezoelectric ultrasound (CVDentus^®^, São Paulo, Brazil) with profuse irrigation of 0.9% sodium chloride solution, the bone blocks containing the implant areas were separated, with a safety margin of 5 mm. The two samples were fixed in 4% buffered formalin for 48 h and sent to the Applied Biotechnology Laboratory (LABA-UFF)—Histology Sector for histological processing following the laboratory protocol.

### 2.9. Laboratory Processing of Samples for Inclusion in Resin

The bone blocks containing the implants were kept in plastic jars containing 4% buffered formaldehyde for 48 h and processed for inclusion in resin without decalcification. After the fixation period, the samples were washed in running water for 24 h and dehydrated under agitation through daily changes of solutions in increasing alcohol concentrations (ethanol 60, 80, 96, and 100%). After dehydration was completed, the samples were subjected to the infiltration process in Technovit resin (7200 VLC, Heraeus Kulzer GmbH & Co., Wehrheim, Germany) through changes every 3 days of solutions containing increasing concentrations of resin (30, 50, 70, and 100%). After the infiltration process, the samples were placed on plastic bases and covered with Technovit resin (7200 VLC, Heraeus Kulzer GmbH & Co., Wehrheim, Germany) to prepare the resin blocks by exposing the set to a light source for polymerization. The blocks containing the implant and peri-implant tissue were glued to previously sanded and cleaned plastic sheets and adapted to the system (EXAKT 300 CP series; Apparatebau, Germany) to be cut in the apical-coronal direction in the longitudinal plane. By cutting the block in the center of the long axis of the implant, it was possible to expose the central surface of the implant. This region was then glued to a second slide to reduce the sample fragment to a final thickness of approximately 30 μm through a polishing protocol using a series of abrasive sandpapers (800, 1000, 1200, and 2400, EXAKT 310 CP series; Apparatebau, Germany) with constant irrigation. The histological sections obtained were stained with Toluidine Blue and covered with a glass coverslip adhered with Entellan^®^ (Merck©, Darmstadt, Germany).

### 2.10. Histomorphometry Analysis

**Figure 2:**
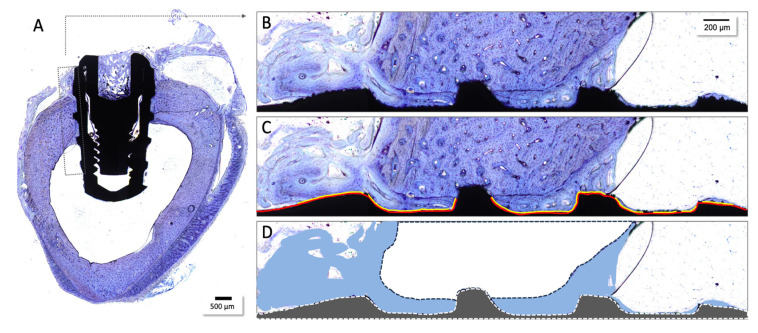
Bone–implant contact (BIC) and bone area fraction occupancy (BAFO) analysis. (**A**) Reconstruction of the area of the implant and adjacent structures. (**B**) Histological reconstruction of the surface of interest marked vertically from the top of the implant to approximately the end of the fourth thread. (**C**) Determination of the line of interest for BIC (red line) in the long implant axis, ignoring the preexistent bone and direct bone–implant contact (yellow line) (total area/BIC) (%). (**D**) For determination of the area of interest for BAFO, the same vertical reference as BIC was used, and, in the horizontal plane, a rectangle with 675 µm width was marked. Areas containing pre-existing bone (white region) and areas containing implant threads (grey regions) were subtracted. The newly formed bone was marked as light blue (total area/BAFO) (%). Stain: Toluidine Blue stained. Scale bar: 500 µm and 200 μm.

Histomorphometric analyses for BIC (bone–implant contact) and BAFO (bone area fraction occupancy) quantification were achieved using an Olympus BX43 light microscope (Tokyo, Japan). Non-superposing photomicrographs with 10× magnification were captured from each histological slide to scan and reconstruct the area of the implant and adjacent structures (Figure 2A). To determine the BIC, the surface of interest was marked vertically from the top of the implant to approximately the end of the fourth thread (Figure 2B). The surface in contact with pre-existing bone was subtracted from the total line (red line), and the new bone in contact with the implant surface was marked as positive (yellow line) (Figure 2C). In the BAFO evaluation, the same vertical reference as BIC was used, and, in the horizontal plane, a rectangle with 675 µm width was marked (Figure 2D). Areas containing pre-existing bone (white region) and areas containing implant threads (grey regions) were subtracted (Figure 2D). The demarcation of BIC and BAFO was conducted using Image J software (LINK: https://imagej.net/ij/, DATE: 12 April 2024) (ImageJ bundled with Java 8) (National Institutes of Health, Bethesda, MD, USA), and the “freehand line” and “freehand selection” tools were manually applied to obtain lines and areas measurements, respectively. Both variables were transformed into a percentage. All histologic slides were coded according to the experimental groups, and an experienced examiner blindly evaluated the slides.

### 2.11. Statistical Analysis

The data were processed using Prism software (v. 10, GraphPad(R)) to prepare descriptive statistics, which included calculating the mean, standard deviation of the mean, and 95% confidence interval. The data showed normal behavior according to the Shapiro–Wilk test. Outliers were not identified using the Rout method with an adjustment of 1%. For inferential analysis, a paired two-tailed Student’s *t*-test was applied to compare the groups. The significance level was set at 5%.

## 3. Results

### 3.1. OES Analysis

**Figure 3:**
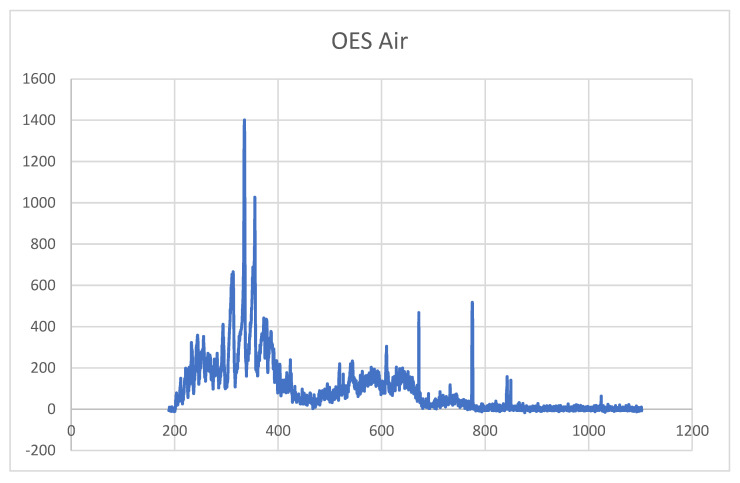
Optical emission spectrum analysis.

Figure 3 shows a representative optical emission spectrum (OES) of the air plasma when the power was fixed at 560 W. The spectrum was acquired in the range from 200 to 1100 nm. This range allows for the detection of emissions from the N2 s positive band, C3Πu-B3Πg, and the N2 first positive band, B3ΠgA3∑u+. It also includes the characteristic lines of highly reactive radicals, such as hydroxyl (OH) at 308 nm and atomic oxygen at 775 and 849 nm. The latter is mainly responsible for the strong oxidation occurring on the titanium surface.

### 3.2. SEM Analyses

**Figure 4:**
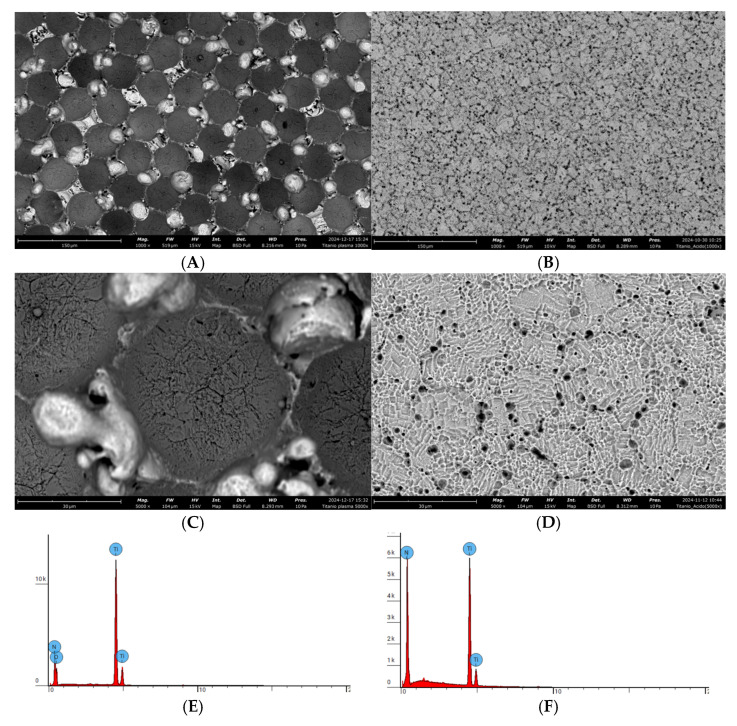
Surface characterization. SEM micrographs of the surfaces of the samples: LS160 1000× (**A**) and 5000× (**C**); SBAE 1000× (**B**) and 5000× (**D**). Scale bar: 30 µm. EDX spectra of LS160 (**E**) and SBAE (**F**).

LS160 (Figure 4A,C) showed a regular pattern with pits of a diameter of about 30 µm contoured by high thin walls characterized by spheric structures (Figure 4C) likely derived from the highly corrosive etching operated by the plasma of air. SBAE (Figure 4B,D) showed a homogeneous microporosity with a distance between the peaks of the order of a few microns, characterized by a high level of cleanliness. Based on the EDX spectra (Figure 4E,F), resulting LS160 was richer in oxygen than SBAE, as could expected from the different manufacturing processes (Table 1).

### 3.3. Protein Adsorption

**Figure 5:**
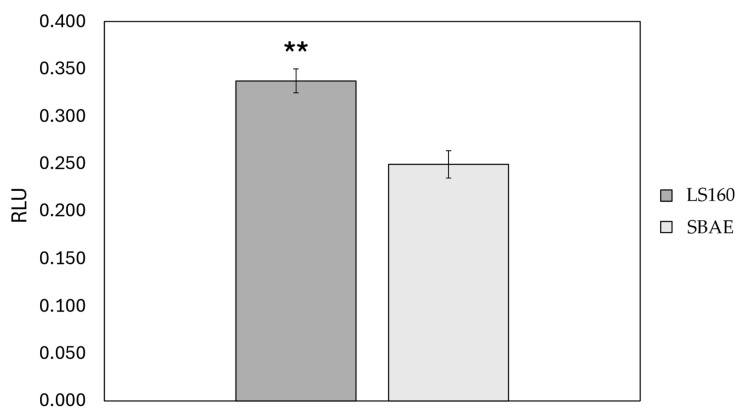
Protein adsorption assay. BSA was used as a protein sample to test the adsorption capability of the surfaces. Standard error is represented with bars, and the level of statistical significance was through ** (*p* < 0.001).

The amount of BSA adsorbed on the LS160 and SBAE surfaces is shown in Figure 5. An increased affinity of BSA for LS160 is apparent and statistically significant.

### 3.4. Cell Morphology

**Figure 6:**
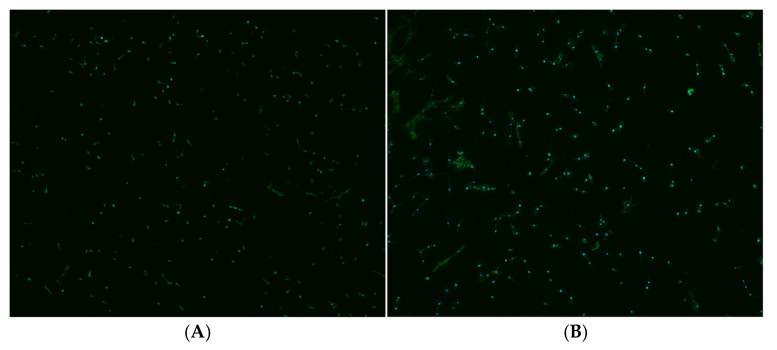

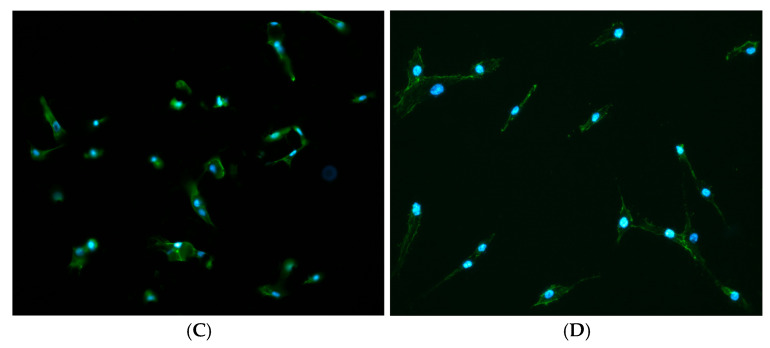
Fluorescent images of the ASCs grown on LS160 4× (**A**) and 20× (**C**) and SBAE 4× (**B**) and 20× (**D**). Nuclei are stained with DAPI, while actin is marked with green, fluorescent phalloidin.

The morphological features of the ASCs grown on the samples at 24 h are reported in Figure 6. The number and the distribution of the adherent cells did not differ in a relevant way on the two surfaces, while some morphological differences may be noted. LS160 surface cells (Figure 6A,C) appear more widespread with broad lamellipodia, while SBAE cells (Figure 6B,D) are more elongated and spindle-shaped. Interestingly, SBAE cells show more evident filopodia and are oriented toward a specific direction, as seen at higher magnification (Figure 6D).

### 3.5. Histological Evaluation

**Figure 7:**
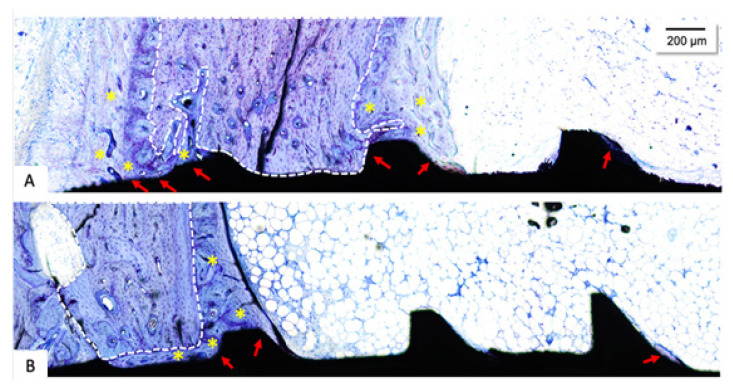
Photomicrographs of adjacent areas from different implant surfaces six weeks after implantation: (**A**) LS160 and (**B**) SBAE. The white dots represent the pre-existing bone. The yellow (*) represents the newly formed bone areas. The red arrows represent regions of bone–implant contact. Stain: Toluidine Blue. Magnification: 10×; Scale bar: 200 μm.

The descriptive histological analysis shows a fraction of preexisting bone in all experimental groups, which probably ensured initial stability. Adjacent to this area, peri-implant bone regeneration can be observed in all tested groups. The histological results of the tested implants are depicted in Figure 7. The LS160 group was surrounded by newly formed bone and broad osteoblastic activity in the region at the top of the implant. A band of newly formed bone was also observed in the medullary space adjacent to the cortical bone. Also, it was possible to observe the bone–implant contact, especially near cortical areas and at the top of the implant (Figure 7A). The SBAE group presented newly formed trabecular bone associated with the preexisting bone area and bone–implant contact, however, in a more discreet way than the previous groups (Figure 7B).

### 3.6. Histomorphometric Analysis

**Figure 8:**
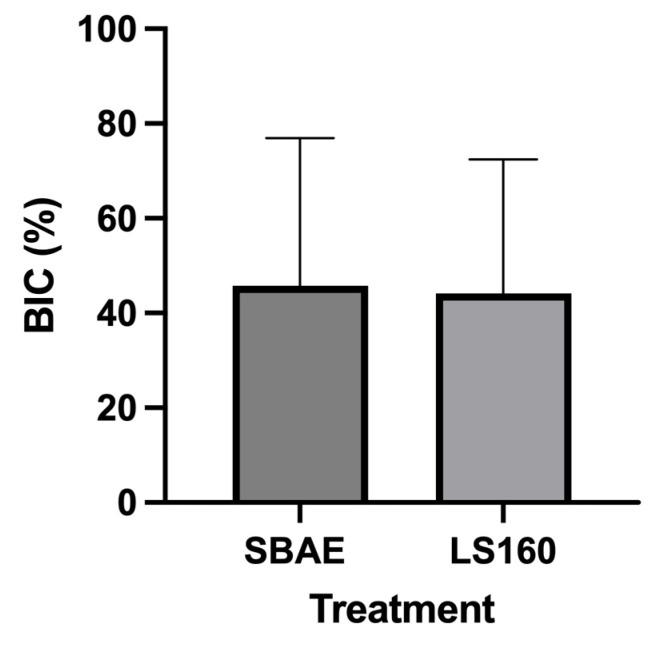
Bone–implant contact (BIC) percentage in the LS160 and SBAE groups six weeks after implantation. Results are presented as mean ±95% confidence interval. After confirming normal distribution with the Shapiro–Wilk test, groups were compared using a paired two-tailed Student’s *t*-test (*p* < 0.05). No statistically significant differences were observed between implant surfaces.

The results of the histomorphometric analysis of BIC and BAFO were considered normal by the Shapiro–Wilk test (*p* > 0.05). Figure 8 shows that after six weeks of implantation, the BIC values for the LS160 and SBAE groups were 44.13 (15.83–72.43) and 39.24 (−10.72–89.21), respectively. The variance analysis (ANOVA and Tukey’s post-test) showed no statistical differences between the tested groups.

**Figure 9:**
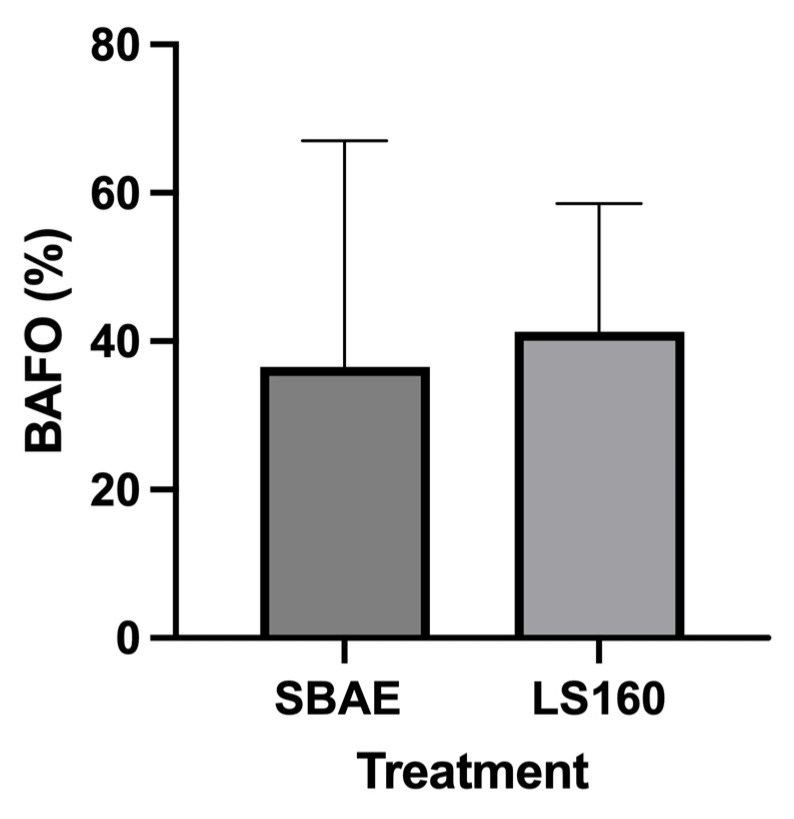
Bone area fraction occupancy (BAFO) percentage in the LS160 and SBAE groups six weeks after implantation. Results are presented as mean ±95% confidence interval. After confirming normal distribution with the Shapiro–Wilk test, groups were compared using a paired two-tailed Student’s *t*-test (*p* < 0.05). No statistically significant differences were observed between implant surfaces.

Similarly, bone volume density showed no statistical differences between groups (ANOVA and Tukey’s post-test), with averages of 41.27 (C.I. 24.00–58.55) and 26.52 (C.I. −17.51–70.54) in the LS160 and SBAE groups, respectively (Figure 9).

## 4. Discussion

The clinical outcome of intra-bony biomaterials has been correlated to the early cell response of bone precursors grown in vitro on their interface [28]. In particular, surface roughness of titanium implants strongly increases cell adhesion, proliferation, and differentiation of osteogenic cells [29,30,31]. Here the authors propose to compare SBAE, a clinical benchmark, to a novel implant surface obtained through laser texturing and plasma treatment, named LS160, with the purpose of assessing its osseointegration potential.

Although SBAE was proven to be very effective in terms of osseointegration, being widely diffused clinically, its preparation process is very laborious and demanding from a chemical point of view. The sulfuric acid-hydrogen peroxide solution required must be prepared each time, as it cannot be stored. Also, its manipulation represents a chemical hazard for the workers, and the waste produced has an environmental impact. The implementation of an alternative treatment with non-inferior biological properties was therefore sought to improve aspects related to the preparation methodology (less polluting), the residual percentage of carbon on the finished surface, and total control of the process, with obvious advantages.

According to the surface characterization performed, LS160 displayed a peculiar microtopography with regular deep pits and high peaks forming an aligned grid, very different from the random pattern achieved by grit blasting and acid etching of SBAE. Consistent with our results depicting a similar number of adherent cells to LS160 and SBAE, Ruffinatti et al. [11] reported that both an aligned pattern with deep pits and a random one with shallow valleys promoted murine osteoblast adhesion more effectively than alternative laser settings. More recently, Melo-Fonseca et al. [32] showed that laser texturing followed by hydrothermal conditioning increased BIC by ~28% in a rabbit femur model, while Henningsen et al. [33] demonstrated that cold atmospheric-plasma functionalization accelerated early bone formation compared with UV activation in a porcine calvarial model. In rodents, a 60 s argon-plasma treatment enhanced reverse-torque values and peri-implant bone density during the first two weeks [34]. Together, these studies reinforce that plasma-assisted or laser-generated micro-topographies can match—or surpass—the osseointegration performance of conventional sand-blasted and acid-etched (SBAE) surfaces while avoiding the occupational hazards and acidic effluents intrinsic to sulfuric-hydrogen-peroxide etching [35,36]. Notably, on LS160, the amount of adsorbed protein increased, in a statistically significant way, compared to SBAE, which is likely due to the enhanced wettability owing to the plasma treatment, a well-known means to augment the surface free energy of a given surface. As a corollary piece of evidence of the plasma jet efficacy, the surface of LS160 resulted in richer oxygen content compared to SBAE (OES and EDX analysis). In addition to the oxygen enrichment, EDX analysis revealed a substantial presence of nitrogen on LS160 (23.87 at%), consistent with the air-based plasma process and the OES results identifying N_2_ emission bands. In contrast, the SBAE surface, treated with argon plasma, showed no detectable nitrogen or oxygen, as expected from the inert nature of the process gas. Our findings therefore support LS160 as a biologically non-inferior and environmentally safer alternative to SBAE.

The rabbit experimental model here adopted is considered suitable for preclinical studies for dental implants evaluation [27] and has advantages such as low cost and similarity in terms of fracture resistance, mineral density, and bone composition [37], in addition to being easy to handle and acquire [38]. Several pre-clinical studies [23,26,27,30,37,39,40] have evaluated the osseointegration of biomaterials and dental implant surfaces in rabbit models after six weeks of implantation. Despite the statistical power of the experimental design, no significant differences in BIC or BAFO were found between the LS160 and SBAE groups. This outcome may be explained by three non-exclusive factors. First, the actual inter-group differences observed were smaller than the effect size used for sample size calculation (<15% vs. 40%), which increases the chance of a type II error. Second, the inherent biological variability of the rabbit tibia model—especially the mixture of cortical and trabecular bone and regional variation—likely introduced heterogeneity that limited discrimination between groups. Third, the six-week healing period may represent a stage at which initial differences in osseointegration kinetics have already converged. Future studies including early and late time-points and larger sample sizes may help clarify these potential effects. The methodology of this study was adapted from the studies by Sartoretto et al. [41], Mello-Machado et al. [42], and Almeida et al. [43], in which no exceptions were made for pre-existing bone areas since the sheep animal model (iliac crest) has predominantly medullary and not cortical bone (not interfering with histological analysis).

A literature review reveals that the rabbit animal model has been widely used in research for decades, ranging from studies to evaluate facial growth patterns [44] to the approach of various anatomical sites for experimentation in the development of biomaterials. Among them, we can mention the femur [25,40,45,46]; intramuscular [47]; calvaria [39]; maxillary sinus [27]; and, very frequently, the tibia [23,24,26,37,48,49,50,51].

The method used for histological evaluation of non-decalcified bone tissue has been widely used in animal model studies for a long time [52]. The methods used to avoid bias in the study included masking [53] of the evaluator for the analysis and interpretation of data without knowledge of the groups tested. Randomization [54] of the positioning of the implants during installation aims to reduce biological and mechanical factors that may interfere with the osseointegration process depending on the region, and only one operator installed all 10 implants. Limitations of this study include the exclusion of samples due to postoperative tibial fracture, the presence of a large amount of cortical bone, and the very inclined positioning of the implant, which prevented a standardized evaluation of the interface according to the methodology used. In addition, there is a limited availability of studies in the literature using rabbit tibias and the same surface treatments to better compare and discuss the results. Prospects include the development of new surfaces to find significant differences, short- and long-term evaluations, clinical validation, testing on lower-density bone models, and subsequently conducting clinical trials.

## 5. Conclusions

Although LS160 and SBAE did not demonstrate statistically significant differences in the osseointegration process during the six-week experimental period, this finding is noteworthy. It suggests that LS160, a surface modification with potentially lower environmental impact, may serve as a viable and more controllable alternative to the traditional clinical benchmark represented by SBAE. Clinicians and manufacturers may consider exploring LS160 as a surface treatment option, especially in contexts where environmental sustainability and process standardization are critical. Further long-term clinical trials are recommended to assess the durability and performance of LS160 beyond the initial healing period across diverse patient populations and anatomical sites. If subsequent research confirms its efficacy and environmental benefits, LS160 may help redefine surface treatment standards in implantology, aligning clinical success with ecological responsibility.

## Figures and Tables

**Table 1:** EDX results showing the atomic (%) and weight (%) concentrations of titanium (Ti), nitrogen (N), and oxygen (O) on the LS160 and SBAE surfaces.

Surface	Ti		N		O	
	at %.	wt %.	at %.	wt %.	at %.	wt %.
**LS160**	57.65	80.3	23.87	8.4	42.35	19.7
**SBAE**	76.13	91.6	-	-	-	-

## Data Availability

The original contributions presented in this study are included in the article. Further inquiries can be directed to the corresponding author.

## Abbreviations

The following abbreviations are used in this manuscript:

ANOVA	Analysis of variance
ASC	Adipose-derived stem cells
BSA	Bovine serum albumin
BAFO	Bone occupied area fraction
BIC	Bone-to-implant contact
CONCEA	Conselho Nacional de Controle de Experimentação Animal
EDX	Energy dispersive X-Ray
SBAE	Sand-blasted acid-etched surface
SEM	Scanning electron microscope
OES	Optical emission spectroscopy
